# Methyl-CpG-binding domain protein 2 epigenetically represses monocyte HLA-DR expression and promotes immune paralysis in HBV-related acute-on-chronic liver failure

**DOI:** 10.3389/fimmu.2026.1694659

**Published:** 2026-02-18

**Authors:** Xiaoqin Liu, Xuhua Jiang, Xueyun Zhang, Qiankun Hu, Xueping Yu, Jian Sun, Mingqin Lu, Jiming Zhang, Yuxian Huang

**Affiliations:** 1 Department of Infectious Diseases, Shanghai Key Laboratory of Infectious Diseases and Biosafety Emergency Response, Shanghai Institute of Infectious Diseases and Biosecurity, National Medical Center for Infectious Diseases, Huashan Hospital, Fudan University, Shanghai, China; 2 Department of Infectious Diseases, The First Affiliated Hospital of Wenzhou Medical University, Wenzhou, Zhejiang, China; 3 Consortium for Infection and Innovation (CII), The First Affiliated Hospital of Wenzhou Medical University, Wenzhou, Zhejiang, China; 4 Clinical Research Center for Liver Diseases, Shanghai Public Health Clinical Center, Fudan University, Shanghai, China; 5 Department of Liver Diseases, Shanghai Public Health Clinical Center, Fudan University, Shanghai, China; 6 Department of Infectious Disease, Clinical Medical Research Center for Bacterial and Fungal Infectious Diseases of Fujian Province, Fujian Medical University Affiliated First Quanzhou Hospital, Quanzhou, Fujian, China; 7 Key Laboratory of Screening and Control of Infectious Diseases (Quanzhou Medical College), Fujian Provincial University, Quanzhou, Fujian, China; 8 Department of Infectious Diseases, The First Affiliated Hospital of Wannan Medical College, Wuhu, China

**Keywords:** acute-on-chronic liver failure, HLA-DR, immune paralysis, methyl-CpG-binding domain protein 2, monocytes

## Abstract

Chronic hepatitis B (CHB) is the leading cause of acute-on-chronic liver failure (ACLF) in China and other Asian countries. A defining immunopathological feature of hepatitis B virus-related ACLF (HBV-ACLF) is immune paralysis, which significantly increases susceptibility to secondary bacterial infections and contributes to poor clinical outcomes. A critical determinant of this immunosuppressed state is impaired antigen presentation due to reduced human leukocyte antigen DR (HLA-DR) expression on monocytes; however, the epigenetic mechanism underlying HLA-DR downregulation in HBV-ACLF remains unclear. Methyl-CpG-binding domain protein 2 (MBD2), an epigenetic reader of DNA methylation, has been implicated in the regulation of monocyte-macrophage function in inflammatory diseases, but its role in HBV-ACLF pathophysiology remains to be fully elucidated. In this study, bulk RNA sequencing (RNA-seq) of circulating monocytes from patients with HBV-ACLF showed a transcriptional profile consistent with immune paralysis, characterized by suppressed antigen presentation and inflammatory pathways, alongside pronounced activation of epigenetic regulatory programs. MBD2 expression was subsequently assessed using immunohistochemistry (IHC), reverse transcription quantitative PCR (RT-qPCR), and flow cytometry. Monocyte MBD2 expression was significantly upregulated in HBV-ACLF and was positively correlated with disease severity (r = 0.2797, P = 0.0182), systemic inflammation indices, and clinical prognosis. To delineate the mechanistic role of MBD2, an MBD2-knockout THP-1 cell model was established and subjected to integrated RNA-seq and assay for transposase-accessible chromatin sequencing (ATAC-seq) following differentiation and lipopolysaccharide (LPS) stimulation. The results showed that MBD2 deficiency significantly increased chromatin accessibility and transcriptional activation of genes involved in antigen presentation and pro-inflammatory responses, including pathways related to major histocompatibility complex (MHC) class II synthesis. Concurrently, enhanced promoter accessibility and activation of transcription factors associated with HLA-II class expression were observed, and increased surface HLA-DR expression was confirmed by flow cytometry. Collectively, these findings suggest that MBD2 epigenetically represses HLA-DR expression in monocytes, leading to impaired antigen presentation and immune paralysis, thereby predisposing patients with HBV-ACLF to secondary bacterial infections. Therefore, MBD2 may serve as a novel biomarker for disease progression and a potential therapeutic target for restoring immunological competence in patients with ACLF.

## 1 Introduction

Acute-on-chronic liver failure (ACLF) is a distinct clinical syndrome characterized by acute hepatic decompensation, intense systemic inflammation, and a high risk of short-term mortality in patients with underlying chronic liver disease (1). In China and other Asian countries, chronic hepatitis B (CHB) remains the primary etiology of ACLF, referred to as HBV-related ACLF (HBV-ACLF) (2). Beyond progressive hepatic dysfunction, ACLF is increasingly recognized as a systemic disorder characterized by profound immune dysregulation. A hallmark of this condition is the development of “immune paralysis,” an acquired immunosuppressive state that closely resembles the immune phenotype observed in severe sepsis (3). Immune paralysis in ACLF is characterized by impaired innate immune responses, compromised antimicrobial defense, and increased susceptibility to secondary infections, all of which significantly contribute to disease severity and mortality (4). Monocytes play a crucial role in mediating this immune dysfunction. Specifically, reduced surface expression of human leukocyte antigen-DR (HLA-DR) on monocytes serves as a critical marker of monocyte deactivation and systemic immunosuppression. Studies have shown that HLA-DR^low^ monocytes exhibit an immunosuppressive phenotype and are strongly associated with secondary infection, disease severity, and poor prognosis following lipopolysaccharide (LPS) stimulation. Functionally, these cells suppress pro-inflammatory responses and attenuate antimicrobial innate immune responses. Notably, restoration of monocyte HLA-DR expression has been shown to partially reverse “sepsis-like” immune paralysis, highlighting monocyte reprogramming as a potential immunomodulatory strategy in ACLF (5–7).

Epigenetic regulation—defined by reversible and heritable modifications that modulate gene expression without altering the primary DNA sequence—has emerged as a critical mechanism regulating immune cell plasticity during systemic inflammation (8). Emerging evidence indicates that monocytes undergo epigenetically driven functional reprogramming during the transition from hyperinflammation to immunosuppression (9). Methyl-binding domain protein 2 (MBD2) has been identified as a critical epigenetic regulator in monocytes (10). Previous studies have shown that MBD2 contributes to disease pathogenesis by inhibiting the expression of key inflammatory and immune-related genes such as Lef-1 and Stat1 (11–13). However, the specific mechanisms driving monocyte immune paralysis in HBV-ACLF and the precise contribution of MBD2 to this process remain poorly understood.

This study aimed to elucidate the epigenetic mechanisms underlying monocyte immune paralysis in HBV-ACLF and to evaluate the potential of MBD2 as both a predictive biomarker and a regulatory factor for immune paralysis in patients with HBV-ACLF.

## 2 Materials and methods

### 2.1 Study population

A total of 71 patients with HBV-ACLF were prospectively recruited from the Department of Infectious Diseases, Huashan Hospital, Fudan University, between December 2018 and January 2021. CHB was diagnosed according to the Guidelines for the Prevention and Treatment of Chronic Hepatitis B (2019 Edition) (14). The diagnosis of HBV-ACLF was established based on the 2019 consensus recommendations of the Asian Pacific Association for the Study of the Liver (APASL) (2). Thirty-nine patients with CHB were included as disease controls, and 25 patients with hepatic hemangioma without underlying liver disease were enrolled as normal controls (NCs). Peripheral venous blood samples (10 mL) were collected from all participants into ethylenediaminetetraacetic acid (EDTA)-anticoagulant tubes. From the three study groups, nine blood samples (n = 3 per group) were randomly selected for bulk RNA sequencing (RNA-seq) analysis. Liver tissue specimens were obtained from patients with HBV-ACLF undergoing liver transplantation (n = 5) and from patients with CHB undergoing percutaneous liver biopsy (n = 5). Normal liver tissues (n = 3) were obtained from non-tumorous liver parenchyma adjacent to hepatic hemangiomas during partial hepatectomy.

Comprehensive clinical and laboratory data were systematically recorded, including demographic characteristics (age and sex), hemoglobin levels, and biochemical indicators of hepatocellular injury and necrosis (total bilirubin, alanine aminotransferase, and aspartate aminotransferase). Systemic inflammatory markers, including white blood cell count, monocyte count, C-reactive protein (CRP), and procalcitonin (PCT), as well as liver coagulation parameters (international normalized ratio [INR]), were also assessed. Disease severity was evaluated using the Model for End-Stage Liver Disease (MELD) score. Bacterial infection was defined according to previously established criteria (15). This study was approved by the Ethics Committee of the Huashan Hospital, Fudan University (approval number 2018-251), and written informed consent was obtained from all participants. Baseline clinical and laboratory characteristics are summarized in **Table 1**.

**Table 1:** Data are presented as median (interquartile range) or number (percentage).

Variable	NC (n = 25)	CHB (n = 39)	HBV-ACLF (n = 71)
Age (years)	37.0 (31.5–47.5)	33.0 (30.0–41.0)	42 (35.5–49.3) **
Male sex, n (%)	13 (52.0)	26 (66.7)	60 (84.5) **
HBsAg (IU/mL)	NA	4911.0 (1033.4–48251.5)	365.6 (250.0–2399.0) **
HBeAg positive, n (%)	NA	25 (64.1)	29 (40.0) *
HBV DNA (log IU/mL)	NA	4.0 (0.0–7.0)	2.0 (2.0–4.0)
Albumin (g/L)	47.5 (42.0–50.3)	48.0 (46.0–50.0)	35.0 (32.0–39.0) **
Total bilirubin	11.3 (9.3–12.5)	14.0 (9.3–18.9)	304.100 (223.1–568.0) **
(μmol/L)			
ALT (U/L)	24.0 (18.0–31.8)	39.0 (24.0–87.0)	91.0 (47.0–279.0) **
AST (U/L)	28.5 (19.0–32.3)	34.0 (21.0–53.0)	99.0 (69.0–217.0) **
White blood cell	4.8 (4.2–6.3)	5.3 (4.6–5.9)	5.4 (3.0–7.4)
count (10^9^/L)			
Neutrophil percentage (%)	55.4 (50.–60.3)	56.2 (49.3–60.7)	73.5 (64.9–84.8) ****
Monocyte count (10^9^/L)	0.36 (0.30–0.50)	0.37 (0.30–0.50)	0.58 (0.40–0.80) **
Hemoglobin	135.0 (125.0–158.0)	158.0 (138.0–166.0)	117.0 (97.5–127.5) **
(g/L)			
Platelets (10^9^	178.0 (109.0–215.0)	206.5 (169.5–243.8)	66.0 (44.0–102.0) **
/L)			
PT (s)	11.9 (11.3–12.5)	12.0 (11.6–12.4)	21.2 (18.6–25.6) **
INR	1.01 (0.98–1.07)	1.05 (1.02–1.08)	1.96 (1.67–2.40) **
PCT (ng/mL)	NA	<0.05	0.54 (0.08–2.00) ****
CRP (mg/L)	NA	6.0 (3.6–7.3)	31.6 (17.3–53.8) ****
LAC (mmol/L)	NA	NA	1.3 (0.8–1.9)
Ascites, n (%)	0	0	41 (57.7)
Hepatic encephalopathy, n (%)	0	0	7 (10.0)
Bacterial infections, n (%)	#0	00	47 (66.2)
Gastrointestinal hemorrhage, n (%)	0	0	6 (8.5)
MELD score	NA	NA	26 (25–28)

NC, normal control; CHB, chronic hepatitis B; HBV-ACLF, hepatitis B virus-related acute-on-chronic liver failure; HbeAg, hepatitis B e antigen; HBV DNA, hepatitis B virus deoxyribonucleic acid; ALT, alanine aminotransferase; AST, aspartate aminotransferase; INR, international normalized ratio; PCT, procalcitonin; CRP, C-reactive protein; LAC, lactic acid; MELD, Model for End-Stage Liver Disease; IU, international unit; NA, not available; *p < 0.05; **p < 0.01; ****p < 0.0001.

### 2.2 Cell isolation and sorting

Peripheral blood mononuclear cells (PBMCs) were isolated from freshly collected EDTA-anticoagulated venous blood by Ficoll–Hypaque density gradient centrifugation (Cedarlane Laboratories, USA). CD14+ monocytes were subsequently purified from the PBMC fraction using magnetic-activated cell sorting with CD14 microbeads (Miltenyi Biotec, Germany). Monocyte purity was assessed by flow cytometry, and only samples with purity exceeding 95% were included for total RNA extraction and downstream transcriptomic analysis.

### 2.3 Assay for transposase-accessible chromatin using sequencing

ATAC-seq was performed to assess genome-wide chromatin accessibility and to identify open regions associated with transcriptional regulation. This method utilizes a hyperactive Tn5 transposase to simultaneously fragment DNA and insert sequencing adapters into regions of accessible chromatin, thereby enabling the mapping of putative transcription factor binding sites and cis-regulatory elements. In this study, ATAC-seq was used to determine whether MBD2 modulates HLA-DR-related gene expression through epigenetic reprogramming of chromatin accessibility.

Six THP-1 cell samples were subjected to ATAC-seq library preparation followed by high-throughput sequencing, yielding a total of 331.53 million high-quality clean reads. Each sample generated a minimum of 50.12 million clean reads, with at least 88.75% of bases achieving a quality score of Q30. Clean reads were aligned to the human reference genome, and peak calling was performed to identify regions of accessible chromatin. Identified peaks were subsequently annotated to nearby genes. Downstream analyses included motif enrichment analysis, transcription factor binding site prediction, and differential peak analysis between experimental groups. Significant differences in chromatin accessibility were further analyzed through functional enrichment analysis.

### 2.4 Bulk RNA-seq

Bulk RNA-seq was conducted on CD14+ monocytes isolated from nine peripheral blood samples, including three NCs, three CHB patients, and three HBV-ACLF patients, matched for age, sex, hepatitis B e antigen (HbeAg) status, and HBV DNA viral load. Following magnetic-activated cell sorting and total RNA extraction, library preparation was performed after rigorous assessment of RNA concentration and integrity. Differential gene expression analysis was performed using the edgeR package (16). Differentially expressed genes (DEGs) were defined by a fold change (FC) ≥ 1.5 and a *p*-value < 0.05, with adjusted *p*-values applied to determine statistical significance. Functional enrichment analyses of DEGs were performed using Gene Ontology (GO)—encompassing biological process (BP), molecular function (MF), and cellular component (CC)—and Kyoto Encyclopedia of Genes and Genomes (KEGG) pathway analyses. In parallel, transcriptomic sequencing was also performed on THP-1 cell samples to enable integrative analysis with the ATAC-seq data and to elucidate the relationship between chromatin accessibility and transcription regulation mediated by MBD2.

### 2.5 Immunohistochemistry

Immunohistochemical analysis was performed to assess the expression level and spatial distribution of MBD2 in liver tissue specimens. Formalin-fixed, paraffin-embedded tissue sections were incubated overnight at 4 °C with a rabbit monoclonal anti-MBD2 antibody (ab188474, 1:200 dilution; Abcam). Following incubation with the appropriate secondary antibodies at room temperature for 1 h, immunoreactivity was visualized using 3,3′-Diaminobenzidine (DAB) as the chromogenic substrate (DAB Detection Kit, Gene Tech, Shanghai, China). MBD2 expression was semi-quantitatively evaluated based on the frequency and intensity of positive staining. The frequency of positively stained cells was scored as follows: 0 (no stained cells), 1+ (weak, <30% positive cells), 2+ (moderate, 30%–60% positive cells), and 3+ (strong, >60% positive cells). Staining intensity was similarly graded as 0 (none), 1+ (weak), 2+ (moderate), or 3+ (strong). The final IHC score was calculated as the sum of the frequency and intensity scores, as previously described (17, 18).

### 2.6 Flow cytometry

Flow cytometry was performed to characterize monocyte subsets and to quantify surface and intracellular expression of immune-related markers in both clinical samples and cell lines. Monocyte gating was conducted according to previously established strategies (19). PBMCs isolated from NC, CHB, and HBV-ACLF patients, as well as THP-1 cells, were labeled with the following fluorochrome-conjugated monoclonal antibodies: APC anti-human HLA-DR (980406, Biolegend, USA), PE/Cy7 anti-human CD16 (302016, Biolegend, USA), and APC/Cy7 anti-human CD14 (#325620, Biolegend, USA). For the detection of nuclear MBD2, cells were fixed and permeabilized using the True-Nuclear ™ Transcription Factor Buffer Set (#424401, BioLegend, USA) according to the manufacturer’s protocol. Cells were then incubated with a rabbit monoclonal anti-MBD2 primary antibody (ab188474, 1:200 dilution; Abcam, UK), followed by staining with an Alexa Fluor^®^ 488-conjugated goat anti-rabbit IgG-H&L secondary antibody (ab150077, dilution 1:2000, Abcam, UK). Fluorescence-minus-one (FMO) controls were used to define gating thresholds for intracellular MBD2 detection, while unstained cells served as blank controls for THP-1 surface marker analysis. Data were acquired on a MoFlo XDP flow cytometer (Beckman, USA) and analyzed using CytExpert (Beckman, USA) and FlowJo software (version 10.8.1; BD, USA).

### 2.7 Cell experiment

Cell culture.

To investigate the mechanisms of monocyte-associated immune paralysis in HBV-ACLF, the human monocytic cell line THP-1 was used to recapitulate the functional characteristics of primary circulating monocytes *in vitro*. THP-1 cell lines were purchased from the Shanghai Institute of Cell Biology, Chinese Academy of Sciences, and maintained in RPMI 1640 medium (#22400-089, Corning, USA) supplemented with 10% fetal bovine serum (FBS; Gibco, USA) and 100 U/ml penicillin-streptomycin (#15070063, Thermo, USA). Cells were cultured at 37 °C in a humidified atmosphere containing 5% CO_2_.

CRISPR/Cas9-mediated knockout of MBD2.

MBD2-knockout THP-1 cells were generated using CRISPR/Cas9 gene-editing technology. Single-guide RNA (sgRNA) sequences targeting the human MBD2 gene were designed using the CRISPOR online tool. The sequences were as follows: forward, 5’-CACCGGAAGCAAGCCTCAGTTGGCA-3’; reverse, 5’-AAACTGCCAACTGAGGCTTGCTTCC-3’.

Annealed sgRNA oligonucleotides were cloned into the lentiCRISPR v2 vector (HanBio, Shanghai, China). THP-1 cells were transduced with the lentiviral construct, and stable knockout cells were established.

Transduced cells were selected with puromycin (4 µg/mL; A1113802, Thermo, USA). As a negative control, THP-1 cells were transduced with the same lentiCRISPR v2 vector carrying a non-targeting sgRNA sequence (CGCTTCCGCGGCCCGTTCAA) and subjected to identical selection procedures. Surviving cells were clonally expanded by limiting dilution. Successful knockout of MBD2 was confirmed at the mRNA and protein levels by reverse transcription quantitative PCR (RT-qPCR) and western blot analysis, respectively.

For subsequent experiments, three THP-1 cell populations were used: wild-type (WT), negative control cells expressing non-targeting sgRNA, and MBD2-/- cells. To induce monocyte activation and an inflammatory state, cells were treated with phorbol-12-myristate-13-acetate (PMA; 50 ng/mL; P1585, Sigma, USA) for 24 h, followed by stimulation with LPS (100 ng/mL; L6386-25MG, Sigma, USA) for 6 h. The cells were then harvested for downstream molecular and functional analyses.

### 2.8 Statistical analysis

Statistical analyses and data visualization were performed using GraphPad Prism 9 (GraphPad Software Inc., San Diego, CA, USA). Continuous variables are presented as medians with interquartile ranges. Inter-group comparisons were conducted using the Kruskal–Wallis test, followed by the Mann–Whitney U test for pairwise comparisons. Categorical variables were compared using the Chi-square test. Correlations between variables were assessed using Spearman’s rank correlation analysis. Bioinformatic analyses, including chromatin accessibility profiling and transcriptomic mapping, were performed using deepTools 3.2.0, DiffBind, the R package ClusterProfiler, and MEME-ChIP for motif enrichment analysis. Figures were prepared using Adobe Photoshop CC 2018. For all analyses, a two-sided *p*-value < 0.05 was considered statistically significant.

## 3 Results

### 3.1 Clinical characteristics

Baseline demographic, clinical, and laboratory data differed significantly between the HBV-ACLF group and the CHB control group. Among the 71 patients with HBV-ACLF, the median age was 42 years (interquartile range: 35.5–49.3), which was significantly higher than that of the CHB group (*p* < 0.01). A strong male predominance was also observed in the HBV-ACLF group (n = 60, 84.5%; *p* < 0.01). Virological profiles revealed significantly lower serum HBsAg levels in HBV-ACLF patients than in CHB controls (*p* < 0.01). Conversely, the proportion of HBeAg–positive patients was higher in the HBV-ACLF group (*p* < 0.05), indicating active viral replication in a significant subset of patients despite advanced hepatic failure.

Biochemical indices demonstrated severe hepatocellular injury and hepatic decompensation in the HBV-ACLF group, evidenced by significantly elevated levels of total bilirubin, alanine aminotransferase (ALT), and aspartate aminotransferase (AST) compared with the CHB group (all *p* < 0.01). Severe coagulopathy was also evident, with significantly higher prothrombin (PT) and INR values (both *p* < 0.01). In contrast, markers of hepatic synthetic function and hematologic parameters, including serum albumin (ALB), hemoglobin concentration, and platelet count (PLT), were significantly lower in the HBV-ACLF group (all *p* < 0.01). Beyond hepatic dysfunction, HBV-ACLF patients exhibited an intense systemic inflammatory response, characterized by a significant increase in circulating monocyte counts (*p* < 0.01), as well as neutrophil percentages, PCT levels, and CRP levels compared with CHB controls (all *p* < 0.0001). Clinical complications observed in the HVB-ACLF group included ascites, hepatic encephalopathy, bacterial infection, and gastrointestinal hemorrhage. Among these complications, bacterial infection was the most prevalent, occurring in approximately 66.2% of patients during hospitalization. The median MELD score for the HBV-ACLF group was 26 (interquartile range: 22–29), reflecting high disease severity and short-term mortality risk. Detailed clinical and laboratory characteristics are summarized in **Table 1**.

### 3.2 Comparative transcriptomic analysis of circulating monocytes in HBV-ACLF patients

**Figure 1:**
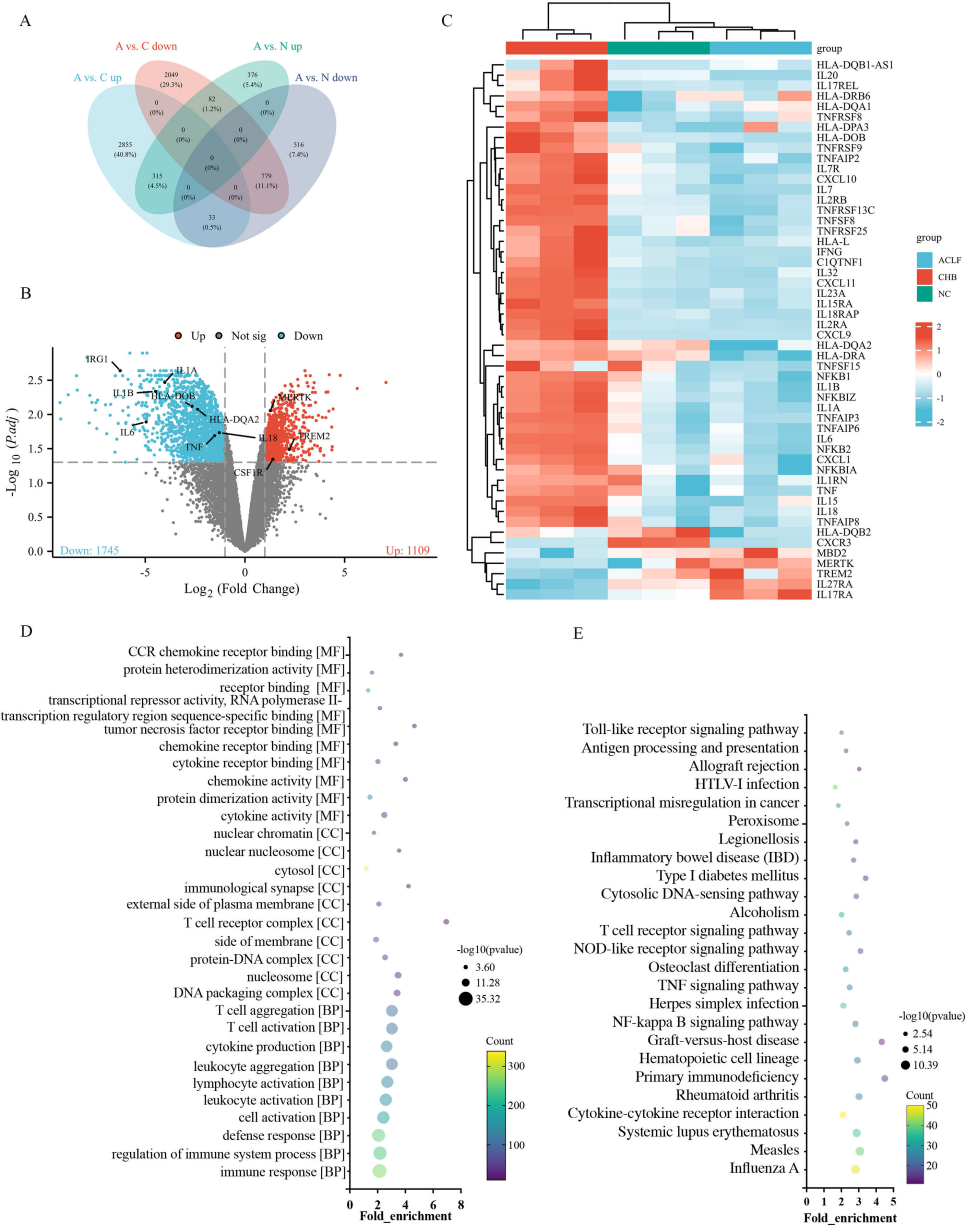
Transcriptomic evidence of monocyte immune paralysis in HBV-ACLF. CD14+ monocytes were isolated by magnetic-activated cell sorting from PBMCs of patients with HBV-ACLF (n = 3), CHB (n = 3), and NCs (n = 3), followed by bulk RNA-seq. **(A)** Venn diagram depicting the overlap and distribution of DEGs among the three study groups; A vs. C represents the comparison between HBV-ACLF and CHB, and A vs. N represents the comparison between HBV-ACLF and NC. **(B)** Volcano plot of DEGs between HBV-ACLF and CHB monocytes, showing significant upregulation of anti-inflammatory and immunoregulatory genes, including MERTK and TREM2, alongside downregulation of key pro-inflammatory mediators and antigen-presentation-related genes, including HLA family members, IL1B, IL6, TNF, and IRG1, consistent with monocyte immune dysfunction. **(C)** Heatmap of inflammation-related genes across the three groups, showing downregulation of pro-inflammatory cytokine (TNF-α, NF-κb, IL1B, IL6, and IL18), chemokine and chemokine receptor (CXCL1, CXCL9, CXCL10, CXCL11, and CXCR3), and MHC class II molecules (HLA-DR, HLA-DQ, and HLA-DP) in HBV-ACLF monocytes, alongside upregulation of anti-inflammatory and immune-regulatory genes (IL17RA, IL27RA, MERTK, TREM2, and MBD2). **(D)** GO enrichment analysis of DEGs between HBV-ACLF and CHB, showing the top 10 enriched MF, BP, and CC terms ranked by fold change, with significant enrichment of inflammatory response, immune regulation, and nuclear chromosome-associated processes. **(E)** KEGG pathway analysis showing significant enrichment of pathways related to innate and adaptive immune signaling, including Toll-like receptor signaling, T cell receptor signaling, antigen processing and presentation, and TNF signaling pathways. NC, normal control; CHB, chronic hepatitis B; HBV-ACLF, acute-on-chronic liver failure related to hepatitis B; DEGs, differentially expressed genes; TNF, tumor necrosis factor; IL, interleukin; MBD2, methyl-binding domain protein 2; GO, Gene Ontology; MF, molecular function; BP, biological process; CC, cellular component; KEGG, Kyoto Encyclopedia of Genes and Genomes.

To delineate monocyte-specific transcriptional changes associated with immune dysregulation in HBV-ACLF, bulk RNA-seq was performed on CD14+ circulating monocytes isolated from HBV-ACLF patients (n = 3), CHB controls (n = 3), and NC (n = 3) (**Supplementary Table 1**). Differential gene expression analysis revealed extensive transcriptomic reprogramming in HBV-ACLF monocytes. Specifically, 3,203 DEGs were significantly upregulated and 2,910 genes were downregulated in the HBV-ACLF group compared with CHB controls. Comparison with the NC group identified 773 upregulated and 1,328 downregulated DEGs. Venn diagram analysis showed distinct DEG signatures between the HBV-ACLF and CHB groups (**Figure 1A**), underscoring significant disease-associated transcriptional reprogramming. To further evaluate the magnitude and biological significance of these changes, an adjusted *p*-value was applied, and differential expression patterns were visualized using volcano plots. The results revealed significant upregulation of genes associated with immunoregulatory and anti-inflammatory functions, including MERTK and TREM2, alongside significant downregulation of pro-inflammatory mediators and antigen presentation genes, including IL1B, IL6, TNF, IRG1, and members of the human leukocyte antigen (HLA) family (**Figure 1B**). Hierarchical clustering and heatmap visualization further delineated this “immune paralysis” signature. Compared with both NC and CHB groups, HBV-ACLF monocytes exhibited significant downregulation of key pro-inflammatory transcripts (TNF-α, NF-ĸb, IL1B, IL6, and IL18), chemokine and chemokine receptors (CXCL1, CXCL9, CXCL10, CXCL11, and CXCR3), and MHC class II molecules (HLA-DR, HLA-DQ, and HLA-DP). In contrast, genes associated with anti-inflammatory signaling and epigenetic regulation—including IL17RA, IL27RA, MERTK, TREM2, and MBD2—were significantly upregulated in the HBV-ACLF group (**Figure 1C**).

To gain functional insight into these transcriptional changes, DEGs identified between HBV-ACLF and CHB were subjected to GO and KEGG pathway enrichment analyses.

GO annotation highlighted the top 10 significantly enriched terms within BP, MF, and CC categories. The analysis revealed significant enrichments of BPs related to inflammatory response, immune activation, and nuclear chromosome activity (**Figure 1D**), indicating widespread epigenetic reprogramming in HBV-ACLF monocytes. Correspondingly, KEGG pathway analysis revealed significant enrichment of pathways relating to innate and adaptive immune regulation, including Toll-like receptor signaling, T cell receptor signaling, antigen processing and presentation, and TNF signaling pathways (**Figure 1E**). Collectively, these transcriptomic profiles suggest a shift toward an immunosuppressive phenotype characteristic of immune paralysis. The concurrent suppression of inflammatory and antigen-presenting pathways, together with enrichment of chromatin-associated processes, suggests epigenetic regulation as a central mechanism underlying monocyte dysfunction in HBV-ACLF. The upregulation of MBD2, a methyl-CpG–binding protein implicated in transcriptional repression and immune cell reprogramming, identifies this factor as a potential regulator of monocyte immune paralysis and a candidate for further mechanistic investigation.

### 3.3 MBD2 expression in HBV-ACLF monocytes is upregulated and is of clinical significance

**Figure 2:**
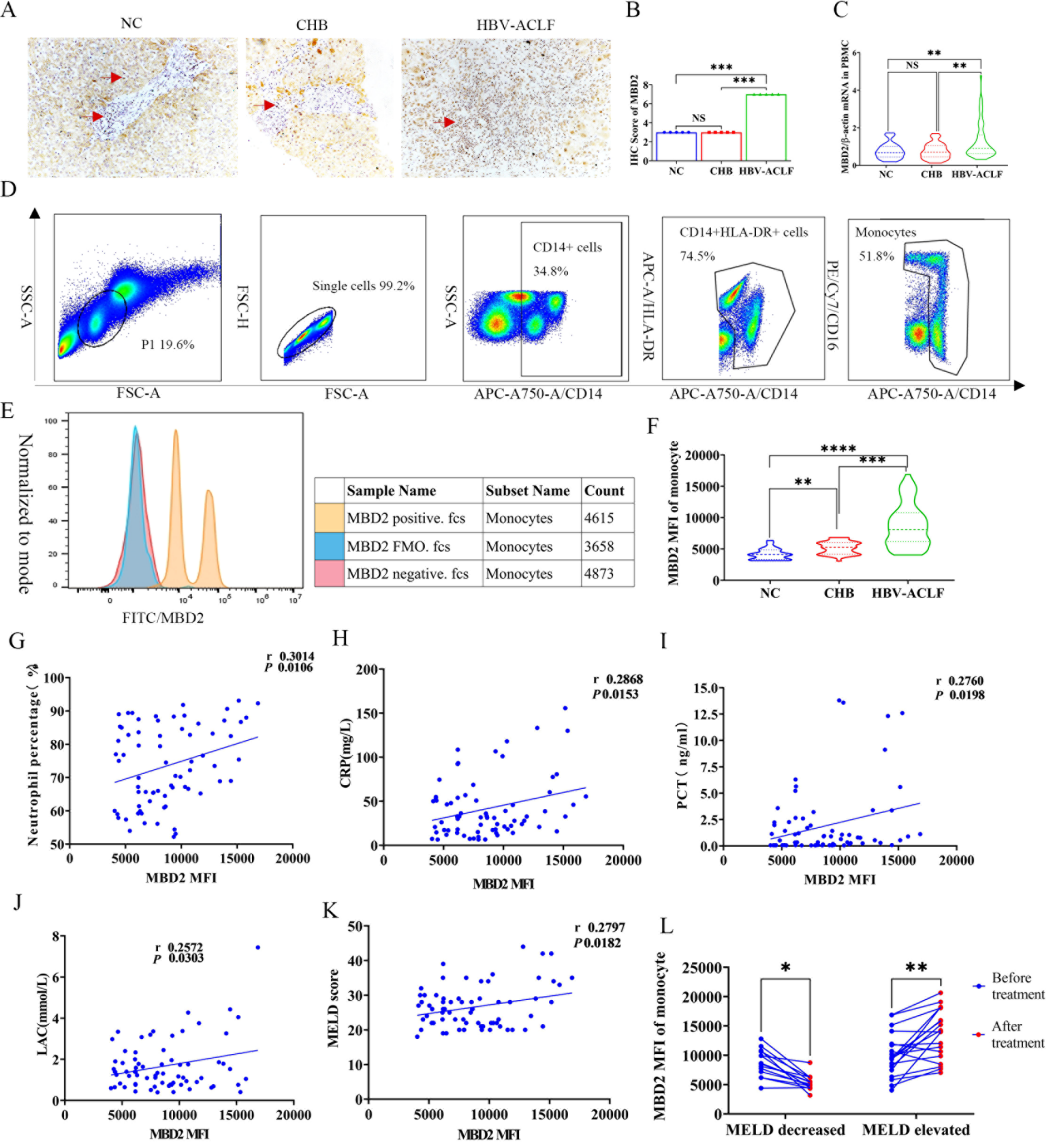
Quantitative analysis of MBD2 expression in HBV-ACLF patients and its clinical significance. MBD2 expression in patients with HBV-ACLF, CHB, and NCs was analyzed using IHC, RT-qPCR, and flow cytometry. **(A)** Representative IHC images (original magnification 200x) showing MBD2-positive cells (indicated with red arrows) predominantly localized within hepatic sinusoids, portal tracts, and inflammatory infiltrates. **(B)** Semi-quantitative analysis of IHC staining based on staining frequency and intensity, showing significantly higher MBD2 expression in the HBV-ACLF group than in the CHB and NC groups. **(C)** RT-qPCR analysis of PBMCs showing significantly higher MBD2 mRNA level in the HBV-ACLF group than in the CHB and NC groups. **(D)** Representative flow cytometric gating strategy for the identification of circulating monocytes and quantification of intracellular MBD2 expression. **(E)** Validation of MBD2 staining specificity and efficiency using MBD2-positive cells, negative controls, and FMO controls. **(F)** Quantitative analysis of MFI showing significantly increased MBD2 protein expression in circulating monocytes from HBV-ACLF patients compared with the CHB and NC groups. **(G–J)** Spearman correlation analyses showing significant positive correlation between monocyte MBD2 MFI and neutrophil percentage, CRP, PCT, and LAC levels in HBV-ACLF patients. **(K)** Positive correlation between monocyte MBD2 expression and MELD score in HBV-ACLF patients. **(L)** Follow-up analysis of 26 HBV-ACLF patients after comprehensive treatment, showing decreased monocyte MBD2 MFI in HBV-ACLF patients with improved disease severity (reduced MELD score) and increased MBD2 MFI in patients with worsening disease (increased MELD score). MBD2, methyl-binding domain protein 2; NC, normal control; CHB, chronic hepatitis B; HBV-ACLF, hepatitis B virus-related acute-on-chronic liver failure; IHC, immunohistochemistry; PBMC, peripheral blood mononuclear cell; FMO, fluorescence-minus-one control; MFI, mean fluorescence intensity; CRP, C-reactive protein; PCT, procalcitonin; LAC, lactic acid; MELD, Model for End Stage Liver Disease. * p < 0.05; ** p < 0.01; *** p < 0.001; **** p < 0.0001.

The expression profile and clinical significance of MBD2 across the HBV-ACLF, CHB, and NC groups were systematically evaluated using IHC, RT-qPCR, and flow cytometry. Immunohistochemical analysis of liver tissue sections revealed the presence of MBD2-positive cells within hepatic sinusoids, portal tracts, and inflammatory infiltrates, consistent with the intrahepatic recruitment and accumulation of circulating monocytes during advanced liver failure (**Figure 2A**). Semi-quantitative analysis of IHC staining frequency and intensity revealed that hepatic MBD2 expression was significantly higher in HBV-ACLF than in both CHB and NC groups (**Figure 2B**). At the transcriptional level, RT-qPCR analysis revealed significantly elevated MBD2 mRNA expression in PBMCs of the HBV-ACLF group compared with the CHB and NC groups (**Figure 2C**). To further define cell-specific protein expression, intracellular MBD2 protein levels in circulating monocytes were quantified by flow cytometry (**Figure 2D**). The results revealed clear separation between MBD2-positive cells, fluorescence-minus-one controls, and negative controls, confirming effective fluorescent staining of MBD2 in monocytes (**Figure 2E**). Quantitative analysis revealed a significantly higher mean fluorescence intensity of MBD2 in monocytes from the HBV-ACLF group than in both control groups (**Figure 2F**). Collectively, these findings indicate that MBD2 is significantly upregulated in HBV-ACLF at both the mRNA and protein levels, particularly within the monocyte compartment.

The clinical relevance of monocyte MBD2 upregulation in HBV-ACLF patients was subsequently analyzed in relation to systemic inflammation and disease severity. In patients with HBV-ACLF, monocyte MBD2 expression was positively correlated with neutrophil percentage (r = 0.3014, *P* = 0.0106), CRP (r = 0.2868, *P* = 0.0153), PCT (r = 0.2760, *P* = 0.0198), and LAC concentration (r = 0.2572, *P* = 0.0303) (**Figures 2G–L**). These associations suggest that MBD2 levels closely reflect the systemic inflammatory and metabolic stress characteristic of HBV-ACLF. In addition, MBD2 expression positively correlated with the MELD score (r = 0.2797, *P* = 0.0182; **Figure 2K**), indicating a strong relationship between MBD2 levels and disease severity. To further assess the clinical relevance of MBD2 expression, changes in MBD2 expression and MELD score were evaluated in 26 patients with HBV-ACLF following comprehensive clinical treatment. The results revealed significantly lower MBD2 expression in HBV-ACLF patients with decreased MELD score (indicating clinical improvement) after treatment than before treatment (*P* = 0.0158), whereas higher MBD2 levels were observed in patients with increased MELD score compared with baseline (*P* = 0.0027; **Figure 2L**). These longitudinal findings suggest that monocyte MBD2 expression dynamically reflects inflammatory activity and disease progression in HBV-ACLF, supporting its potential utility as a biomarker of immune dysfunction and disease severity.

### 3.4 Epigenetic mechanism of MBD2-mediated immune paralysis

**Figure 3:**
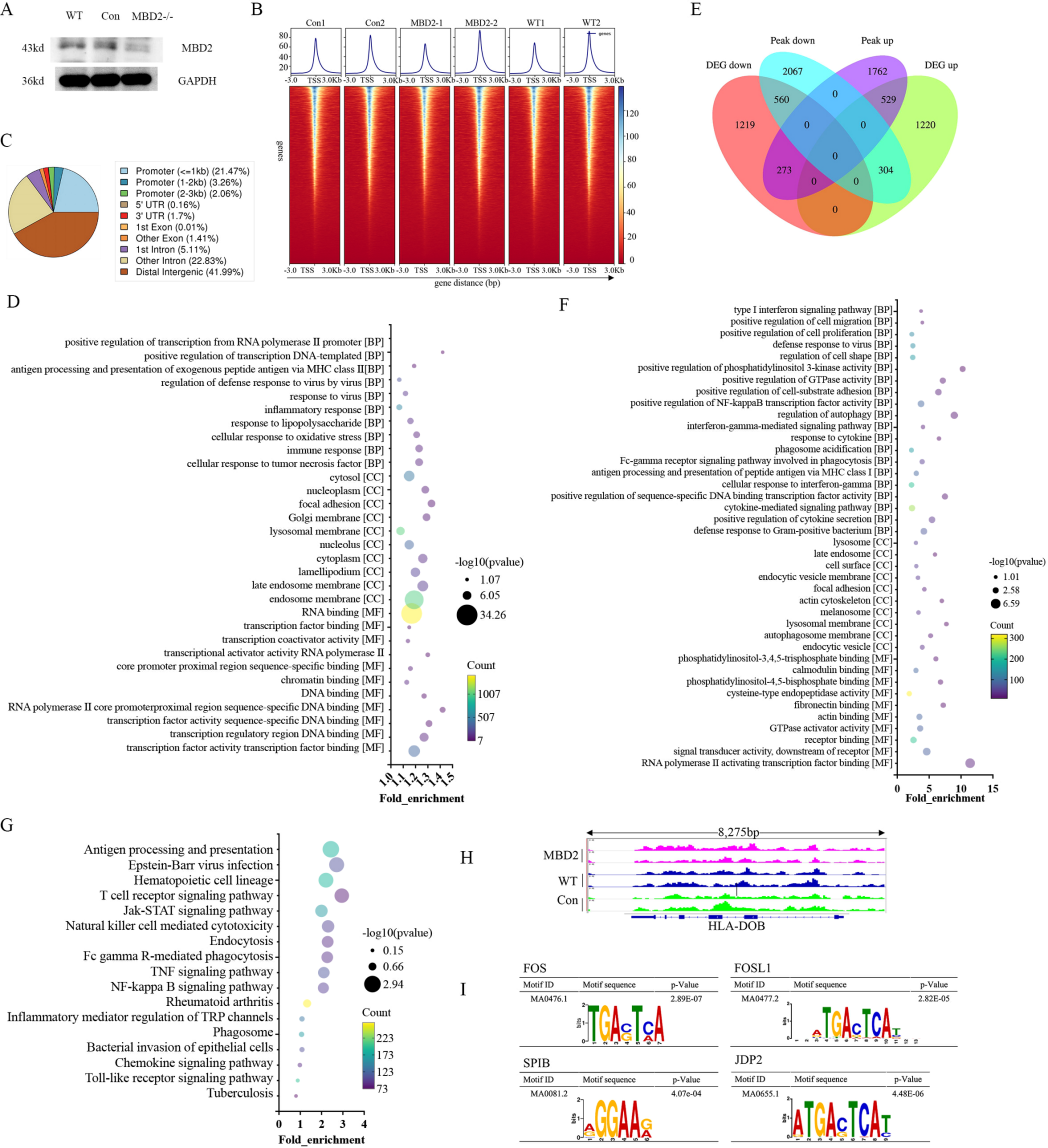
Epigenetic mechanisms underlying MBD2-mediated immune paralysis. **(A)** Western blot analysis confirming efficient CRISPR-Cas9-mediated MBD2 knockout in the THP-1 cell line. MBD2-/-, WT, and negative control THP-1 cells were subjected to PMA-induced differentiation, followed by immune tolerance-like stimulation, and subsequently analyzed using integrated RNA-seq and ATAC-seq. **(B)** Heatmap of ATAC-seq signal distribution within 3 kb of the TSS, demonstrating significant enrichment near the TSS and indicating high-quality chromatin accessibility profiling. **(C)** Genomic annotation of ATAC-seq peaks showing enrichment within promoter regulatory regions. **(D)** GO enrichment analysis of DARs, showing an upregulation of antigen-presentation and pro-inflammatory pathways in MBD2-/- cells, consistent with the attenuation of immune paralysis. **(E)** Venn diagram depicting the overlap between DARs and DEGs identified by integrated RNA-seq and ATAC-seq. **(F)** KEGG pathway enrichment analysis showing increased activation of phagocytosis, antigen processing and presentation, and Toll-like receptor signaling in THP-1 MBD2-/- cells compared with controls. **(G)** GO enrichment analysis confirming upregulation of antigen presentation-related and pro-inflammatory BPs. **(H)** Differential peak analysis showing significantly increased chromatin accessibility at the HLA-DOB promoter region in THP-1 MBD2-/- cells compared with the control. **(I)** Motif enrichment analysis of DARs showing significantly increased representation of transcription factor binding motifs, including FOS, FOSL1, SPIB, and JDP2—implicated in antigen presentation and processing, inflammatory signaling, and immune cell activation—in the THP-1 MBD2-/- group. MBD2, methyl-binding domain protein 2; ATAC-seq, assay for transposase-accessible chromatin with high-throughput sequencing; RNA-seq, RNA sequencing technology; WT, wild type; TSS, transcription start site; GO, Gene Ontology; KEGG, Kyoto Encyclopedia of Genes and Genomes; BP, biological process; MF, molecular function; CC, cellular component; DARs, differentially accessible regions; DEGs, differentially expressed genes.

Given the strong correlation between monocyte MBD2 expression, systemic inflammation, and disease severity in HBV-ACLF patients, the functional role of MBD2 in regulating monocyte immune responses was further investigated at both transcriptional and epigenetic levels. THP-1 cells with MBD2 knockout were established using CRISPR/Cas9-mediated gene editing and validated by western blot analysis (**Figure 3A**). To simulate the inflammatory and immune-tolerant milieu characteristic of ACLF, MBD2-/-, WT, and negative control THP-1 cells were differentiated with PMA (100 ng/mL) and subsequently stimulated with LPS (100 ng/mL). Integrated transcriptomic and chromatin accessibility profiling was then performed using RNA-Seq and ATAC-Seq.

Quality assessment of ATAC-seq data demonstrated robust enrichment of sequencing reads within 3 kb of transcription start sites (TSSs) across all samples, confirming high data quality and accurate profiling of open chromatin regions (**Figure 3B**). Genome-wide peak calling identified accessible chromatin regions, which were subsequently annotated to the nearest genes. A significant proportion of these peaks was localized to promoter regions, facilitating the functional interpretation of transcriptional regulatory elements (**Figure 3C**).

Comparative analyses revealed that MBD2 deficiency significantly altered immune-related transcriptional programs. Compared with the control group, MBD2-knockout THP-1 cells exhibited significant enrichment of BPs associated with antigen processing and presentation through MHC class II, antiviral defense responses, inflammatory responses, and cellular response to LPS. In parallel, MF terms related to transcriptional regulation and sequence-specific DNA binding were also significantly altered, consistent with broad epigenetic reprogramming (**Figure 3D**). To identify genes directly regulated by MBD2-mediated chromatin remodeling, differentially accessible regions (DARs) identified by ATAC-seq were intersected with DEGs derived from RNA-seq. Venn diagram analysis revealed a significant overlap between genes exhibiting increased chromatin accessibility and transcriptional upregulation following MBD2 deletion, supporting a direct regulatory role for MBD1 (**Figure 3E**).

Functional annotation of these overlapping gene sets by GO enrichment and KEGG pathway analyses demonstrated a consistent shift toward immune activation.

Specifically, THP-1 MBD2-/- cells showed significantly increased capacities for Fc-gamma receptor-mediated phagocytosis, antigen processing and presentation through both MHC class I and class II pathways, interferon-gamma-mediated responses, positive regulation of sequence-specific DNA binding transcription factor activity, cytokine signaling, and positive regulation of cytokine secretion (**Figure 3F**). Consistently, KEGG pathway analysis further showed significant enrichment of innate and adaptive immune signaling pathways, including endocytosis, Fc-gamma receptor-mediated phagocytosis, TNF signaling, NF-κB signaling, chemokine signaling, and Toll-like receptor signaling pathways (**Figure 3G**). Integrative analysis of ATAC-seq and RNA-seq data identified MBD2 as a negative regulator of MHC class II synthesis and and antigen-presenting immune functions (**Figures 3D, G**). Notably, chromatin accessibility at the HLA-DOB promoter—a critical regulator of the HLA-DR peptide loading and surface expression—was significantly increased in THP-1 MBD2-/- cells compared with controls (**Figure 3H**). Furthermore, motif enrichment analysis of DARs revealed overrepresentation of binding motifs for key transcription factors, including FOS, FOSL1, SPIB, and JDP2, essential for antigen presentation and pro-inflammatory gene transcription (**Figure 3I**). Collectively, these findings suggest that MBD2 epigenetically limits chromatin accessibility at HLA class II loci and associated inflammatory pathways. MBD2 deficiency restores transcriptional expression by modulating the chromosomal accessibility and immune effector programs, supporting the hypothesis that MBD2-mediated epigenetic repression of HLA-DR and related antigen-presentation machinery drives the immune paralysis phenotype observed in monocytes from patients with HBV-ACLF.

### 3.5 MBD2 induces immune paralysis by negatively regulating HLA-DR expression on monocytes

**Figure 4:**
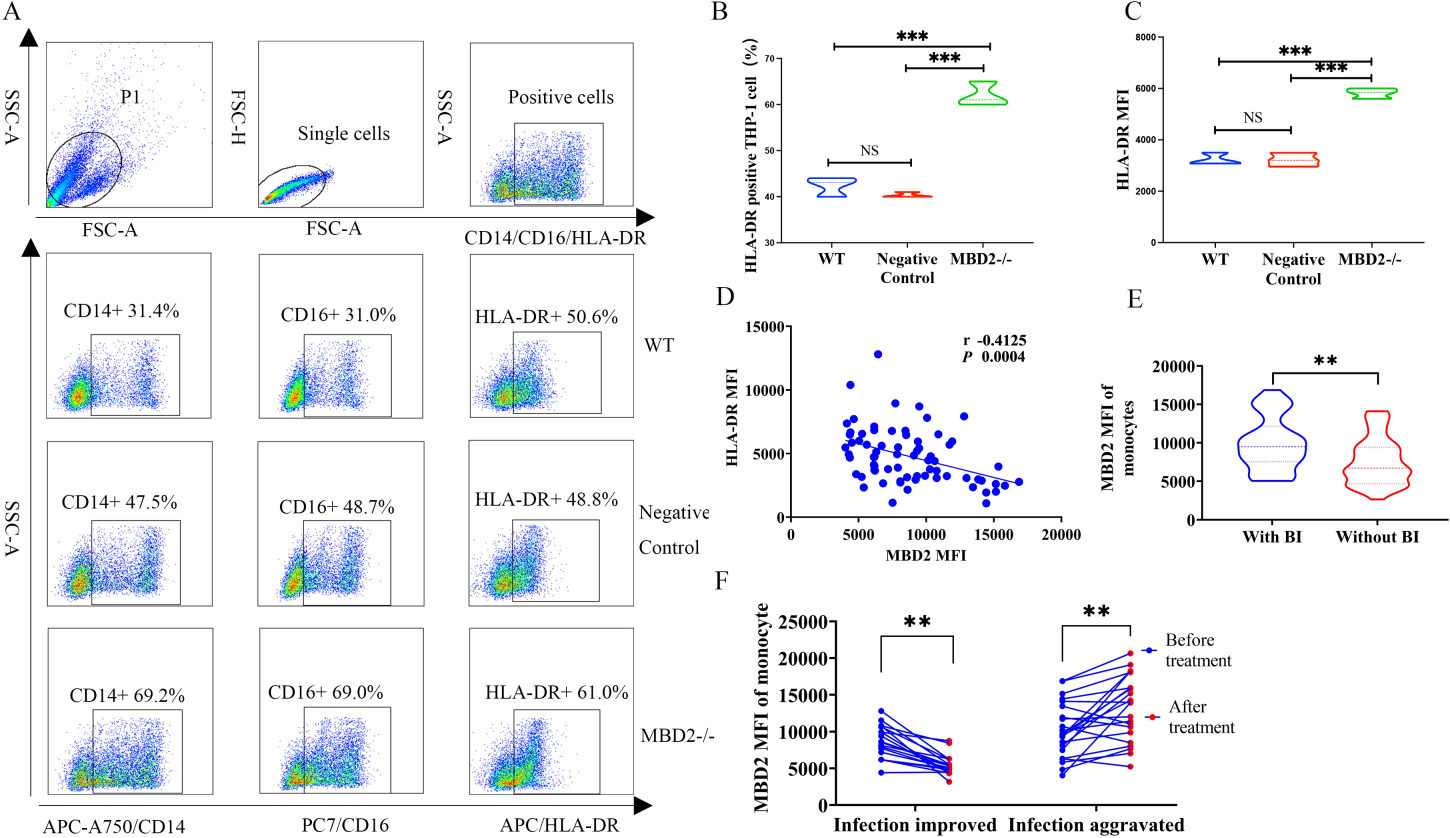
MBD2 negatively regulates HLA-DR expression on monocytes and promotes immune paralysis. To validate the inhibitory effect of MBD2 on monocyte HLA-DR expression, surface expression of CD14, CD16, and HLA-DR was analyzed in THP-1 cells by flow cytometry. Unstained cells were used as blank controls to assess background autofluorescence. **(A)** Representative flow cytometric analysis showing significantly higher proportions of CD14+, CD16+, and HLA-DR+ cells in THP-1 MBD2-/- cells than in WT and negative controls. **(B)** Quantitative analysis of the percentages of HLA-DR-positive cells across the three groups, showing a significant increase in THP-1 MBD2-/- cells compared with WT and negative control cells. **(C)** Quantitative analysis of HLA-DR MFI, confirming significant upregulation of surface HLA-DR expression in THP-1 MBD2-/- cells compared with WT and negative control cells. **(D)** Correlation analysis showing a significant negative correlation between MBD2 expression and HLA-DR expression in monocytes, suggesting an inhibitory role of MBD2 in monocyte antigen-presenting capacity. **(E)** Comparison of monocyte MBD2 MFI in HBV-ACLF patients with and without bacterial infection, showing significantly increased MBD2 expression in patients with bacterial infection compared with those without bacterial infection. **(F)** Longitudinal follow-up in 21 HBV-ACLF patients after comprehensive clinical intervention, showing a significant decrease in monocyte MBD2 MFI in patients with improvement of infection after treatment, whereas increased MBD2 MFI was observed in patients with aggravated or refractory infections. MBD2, methyl-binding domain protein 2; WT, wild type; MFI, mean fluorescence intensity; BI, bacterial infection; NS, no significant difference; HBV-ACLF, hepatitis B virus-related acute-on-chronic liver failure. ** p < 0.01; *** p < 0.001.

To functionally validate the inhibitory role of MBD2 on monocyte antigen-presenting capacity, surface HLA-DR expression was assessed in THP-1 cells by flow cytometry. Compared with WT and negative control cells, THP-1 MBD2-/- cells exhibited significantly increased proportions of CD14+ cells (69.2% vs. 31.4% and 47.5%, respectively), CD16+ cells (69.0% vs. 31.0% and 48.7%), and HLA-DR+ cells (61.0% vs. 50.6% and 48.8%) (**Figure 4A**). Consistent with these findings, quantitative analysis of MFI demonstrated that HLA-DR expression was significantly higher in THP-1 MBD2-/- cells than in both control groups (**Figure 4C**), confirming enhanced HLA-DR surface expression following MBD2 depletion. Correlation analyses further revealed significant negative correlations between MBD2 and HLA-DR expression levels in circulating monocytes, as determined by flow cytometry (**Figure 4D**), reinforcing the hypothesis that MBD2 functions as a negative regulator of HLA-DR expression. These findings support an epigenetic mechanism through which elevated MBD2 expression suppresses monocyte antigen-presenting capacity by downregulating HLA-DR. To determine the clinical relevance of monocyte MBD2 expression in HBV-ACLF, the expression levels of MBD2 MFI in HBV-ACLF patients with (n = 33) and without (n = 38) bacterial infection were evaluated. The results showed that HBV-ACLF patients with concurrent bacterial infection exhibited significantly higher monocyte MBD2 MFI than those without infection (9490 vs. 6575, *P* = 0.0025; **Figure 4E**), indicating a strong association between elevated MBD2 expression and increased susceptibility to secondary infection. To further assess the dynamic relationship between MBD2 expression and infection status, longitudinal analyses were conducted in 21 HBV-ACLF patients before and after comprehensive treatment. The results showed that patients with clinical improvement of infection exhibited a significant reduction in MBD2 MFI compared with pretreatment levels (8749 vs. 5350, *P =* 0.0158). In contrast, patients with aggravated or refractory infections showed a significant increase in MBD2 MFI (10032 *vs.* 13602*, P =* 0.0027; **Figure 4F**). Collectively, these findings suggest that MBD2 suppresses HLA-DR expression on monocytes, thereby impairing antigen presentation and promoting an immunoparalytic phenotype. This epigenetically mediated immune dysfunction is closely associated with increased risk of secondary infection, disease severity, and poor clinical prognosis in HBV-ACLF, highlighting MBD2 as both a mechanistic driver of immune paralysis and a potential predictive biomarker of immunological failure.

## 4 Discussion

Bacterial infection is a frequent and early complication of ACLF that markedly affects disease progression and mortality. Previous studies and our data consistently show a high incidence of early secondary infections, supporting the presence of immune paralysis characterized by ineffective antimicrobial immunity (3, 15, 20). Effective antimicrobial immunity critically depends on intact antigen presentation. HLA-DR is a highly expressed and functionally essential MHC class II molecule in antigen presentation, with a complex genetic architecture encoded by multiple loci and functionally distinct genes (21). By presenting processed antigens to T cell receptors, HLA-DR-expressing monocytes initiate and sustain adaptive immune responses against invading pathogens (22). Genetic and functional studies have identified HLA-DR as a primary locus for susceptibility to HBV-ACLF, linking reduced HLA-DR expression to impaired innate immune responses, increased susceptibility to secondary infections, disease severity, and poor prognosis (5, 7). Notably, the immune status in ACLF is characterized by decreased HLA-DR expression on circulating monocytes and a decreased capacity of monocytes and macrophages to release pro-inflammatory cytokines upon stimulation. This immunosuppressed state closely resembles the immune paralysis observed in sepsis and is considered a central mechanism predisposing patients to secondary infection (3).

Consistent with this paradigm, monocyte bulk RNA-seq in the present study revealed a transcriptomic signature of immune paralysis in HBV-ACLF, characterized by the downregulation of HLA-DR and proinflammatory cytokines, thereby providing transcriptomic evidence of impaired antimicrobial innate immune responses. In sepsis, this state of immune paralysis is often referred to a state of hyporesponsiveness to LPS challenge following an initial inflammatory insult (23). This phenomenon is highly relevant to ACLF pathophysiology. In ACLF, increased intestinal permeability and gut microbiota dysbiosis facilitate the translocation of LPS into systemic circulation, driving persistent systemic inflammation even in the absence of overt infection (24). Sustained exposure to these microbial products may induce a compensatory, epigenetically regulated immunosuppressive program, ultimately culminating in monocyte dysfunction and immune paralysis (25). However, despite the recognized importance of epigenetic regulation in modulating innate immune responses, the specific epigenetic modifiers and chromatin remodeling mechanisms underlying suppression of inflammatory genes, including HLA-DR, in HBV-ACLF remain elusive.

MBD proteins function as key epigenetic readers that interpret DNA methylation signals and translate them into context-dependent transcriptional outcomes (12). In recent years, this protein family has attracted increasing attention due to its therapeutic potential in immune-mediated inflammatory disorders (12, 26). Among the MBD proteins, MBD2 is particularly promising, as genetic deletion of MBD2 in murine models does not result in overt developmental abnormalities or major deleterious physiological effects, supporting its suitability as a potential therapeutic target (27). Functionally, MBD2 has been identified as a key inhibitory transcription factor in monocytes, where it modulates immune gene expression programs through epigenetic mechanisms (10). Previous studies have shown that MBD2 contributes to the pathogenesis of inflammatory diseases by regulating immune cell activation, polarization, and effector functions (13, 28, 29). In the present study, flow cytometric analyses revealed that monocyte MBD2 expression significantly correlated with systemic inflammatory activity and disease severity in HBV-ACLF patients. These findings extend previous mechanistic research by directly linking MBD2 expression to immune dysregulation in a clinically relevant setting. Previous studies have demonstrated that MBD2 selectively binds to methylated promoters of immune-related genes in macrophages and monocytes, leading to transcriptional repression and influencing altered immune phenotype (13, 28, 29). Consistently, monocytes derived from Mbd2^-/-^ mice exhibit enhanced MHC class II synthesis, increased antigen-presenting capacity, heightened inflammatory responsiveness, and improved bactericidal activity (10). Together with the present findings, these observations suggest that MBD2 exerts a potent negative epigenetic regulatory effect on the proinflammatory and antimicrobial programs in monocytes and macrophages.

To further delineate the underlying molecular mechanisms, a THP-1 MBD2-/- cell model was established and subjected to integrated RNA-seq and ATAC-seq analysis following PMA-induced differentiation and LPS stimulation. This combined approach enabled simultaneous evaluation of transcriptional changes and chromatin accessibility landscapes associated with MBD2 deficiency. GO enrichment and KEGG pathway analyses of promoter-associated DARs revealed broad activation of antigen-presentation pathways and pro-inflammatory signaling cascade in THP-1 MBD2-/- cells. These findings indicate that relief of MBD2-mediated transcription repression restores monocyte antimicrobial defense programs and reverses key features of immune paralysis at the genome-wide level. This reversal was primarily attributed to changes in chromatin accessibility within the HLA locus. Specifically, the results suggest that MBD2 modulates the transcription of HLA-DOB, a non-classical MHC class II molecule. This finding is particularly significant, as Castelli et al. have reported that HLA-DOB may directly influence antigen presentation in the MHC class II pathway (30). As the most abundantly and consistently expressed MHC class II molecule on human monocytes, HLA-DR serves as a key functional indicator of antigen-presenting capacity (31). In sepsis and ACLF, reduced monocyte HLA-DR expression is a well-established hallmark of immune paralysis and is closely associated with secondary infection and adverse clinical outcomes (3, 31). Consistent with this paradigm, flow cytometric analyses in the present study demonstrated a significant negative correlation between MBD2 expression and HLA-DR levels in both THP-1 cells and primary monocytes from patients with HBV-ACLF. Furthermore, changes in MBD2 expression were closely associated with infection status and prognosis; specifically, higher MBD2 levels were observed in patients with bacterial infections and worsening clinical trajectories. Collectively, these findings provide functional and clinical evidence that MBD2-mediated repression of HLA-DR contributes to monocyte dysfunction, immune paralysis, and increased susceptibility to secondary infection in HBV-ACLF.

## 5 Conclusion

This study provides the first evidence that MBD2 expression is significantly upregulated in circulating monocytes of patients with HBV-ACLF and that elevated MBD2 levels are closely associated with inflammatory burden, disease severity, and infection risk.

By integrating RNA-seq and ATAC-seq, the epigenetic mechanism by which MBD2 suppresses antimicrobial innate immune responses was elucidated. Specifically, MBD2 was shown to negatively regulate HLA-DR expression, leading to impaired antigen presentation and attenuated pro-inflammatory signaling, a phenotype consistent with immune paralysis in HBV-ACLF. This regulatory effect is likely mediated, at least in part, through epigenetic regulation of HLA-DOB-associated transcription in monocytes. Collectively, these findings highlight MBD2 as both a potential biomarker for immune dysfunction and a therapeutic target for restoring monocyte immune competence in HBV-ACLF. Targeting MBD2-mediated epigenetic repression may represent a novel strategy for reversing immune paralysis, reducing secondary infections, and improving clinical outcomes in patients with HBV-ACLF.

This study has several limitations. Functional pharmacological inhibition of MBD2 was not performed, and chromatin immunoprecipitation sequencing is required to provide direct evidence of MBD2 binding at the HLA-DOB promoter. In addition, validation of these findings in an Mbd2^-/-^ ACLF animal model is necessary to establish a causal relationship between MBD2-mediated MHC class II inhibition, immune paralysis, and secondary infection *in vivo*.

## Data Availability

The datasets presented in this article are not readily available because the corresponding ethical documents do not include the public release of transcriptome sequencing information. Requests to access the datasets should be directed to the correponding author, Yuxian Huang [yxhuang@fudan.edu.cn].
