# Supplementary material for: Methyl-CpG-binding domain protein 2 epigenetically represses monocyte HLA-DR expression and promotes immune paralysis in HBV-related acute-on-chronic liver failure
Source: Front Immunol. 2026 Feb 18;17:1694659. doi: 10.3389/fimmu.2026.1694659 (PMC12956542; doi:10.3389/fimmu.2026.1694659)
Supplement: Supplementary file 2 [file Table1.docx]

Supplementary table1. Clinical baseline data of RNA sequencing analysis

|  | NC(n=3) | CHB(n=3) | HBV-ACLF(n=3) |
| --- | --- | --- | --- |
| Age (yr) | 38 ± 6 | 39 ± 5 | 47 ± 3 |
| Male sex, n(%) | 2(66.7%) | 2(66.7%) | 2(66.7%) |
| HBsAg (IU/ml) | NA | 14359.3 ±12007.9 | 5015.9 ± 4246.6 |
| HBeAg positive，n(%) | NA | 2(66.7%) | 1(33.3%) |
| HBV DNA (Lg IU/ml) | NA | 4 ± 4 | 2 ± 2 |
| Total bilirubin (µmol/L) | 10.9 ± 1.2 | 14.1 ± 2.7 | 451.4 ± 238.6 |
| Albumin (g/L) | 49.3 ± 3.1 | 46.7 ± 4.0 | 32.7 ± 9.5 |
| ALT(IU/L) | 21.3 ± 3.8 | 45.0 ± 38.1 | 134.7 ± 124.5 |
| AST(IU/L) | 23.7 ± 8.1 | 33.3 ± 17.5 | 102.0 ± 61.9 |
| White blood cell count (10^9^/L) | 5.7 ± 1.0 | 6.2 ± 1.9 | 6.0 ± 2.0 |
| Monocyte count (10^9^/L) | 0.25 ± 0.07 | 0.42 ± 0.11 | 0.67 ± 0.21 |
| INR | 0.98 ± 0.02 | 1.04 ± 0.06 | 2.29 ± 0.40 |
| MELD score | NA | NA | 28 ± 4 |

Supplementary table1. Clinical baseline data of RNA sequencing analysis. Data are expressed as mean and standard deviation, or number (percent). NC: normal control; CHB: Chronic Hepatitis B; HBV-ACLF: Hepatitis B virus-related acute-on-chronic liver failure; HBeAg: Hepatitis Be Antigen; HBV DNA: Hepatitis B Virus Deoxyribonucleic Acid; ALT: Alanine aminotransferase; AST: aspartate aminotransferase; INR: international normalized ratio; MELD, Model for End Stage Liver Disease; IU: international unit; NA: not available
